# Recent Advances in Porous Bio-Polymer Composites for the Remediation of Organic Pollutants

**DOI:** 10.3390/polym16111543

**Published:** 2024-05-30

**Authors:** Nayereh S. Tadayoni, Mohammad Dinari, Aleena Roy, Mahmood Karimi Abdolmaleki

**Affiliations:** 1 Department of Chemistry, Isfahan University of Technology, Isfahan 84156-83111, Iran; nayereh1370@yahoo.com; 2 Department of Physical and Environmental Sciences, Texas A&M University-Corpus Christi, Corpus Christi, TX 78412, USA

**Keywords:** porous biopolymer, organic pollutants, wastewater treatment

## Abstract

The increasing awareness of the importance of a clean and sustainable environment, coupled with the rapid growth of both population and technology, has instilled in people a strong inclination to address the issue of wastewater treatment. This global concern has prompted individuals to prioritize the proper management and purification of wastewater. Organic pollutants are very persistent and due to their destructive effects, it is necessary to remove them from wastewater. In the last decade, porous organic polymers (POPs) have garnered interest among researchers due to their effectiveness in removing various types of pollutants. Porous biopolymers seem to be suitable candidates among POPs. Sustainable consumption and environmental protection, as well as reducing the consumption of toxic chemicals, are the advantages of using biopolymers in the preparation of effective composites to remove pollutants. Composites containing porous biopolymers, like other POPs, can remove various pollutants through absorption, membrane filtration, or oxidative and photocatalytic effects. Although composites based on porous biopolymers shown relatively good performance in removing pollutants, their insufficient strength limits their performance. On the other hand, in comparison with other POPs, including covalent organic frameworks, they have weaker performance. Therefore, porous organic biopolymers are generally used in composites with other compounds. Therefore, it seems necessary to research the performance of these composites and investigate the reasons for using composite components. This review exhaustively investigates the recent progress in the use of composites containing porous biopolymers in the removal of organic pollutants in the form of adsorbents, membranes, catalysts, etc. Information regarding the mechanism, composite functionality, and the reasons for using each component in the construction of composites are discussed. The following provides a vision of future opportunities for the preparation of porous composites from biopolymers.

## 1. Introduction

The availability of clean water guarantees the health and well-being of living beings [1]. Population growth and industrialization are two influential factors in increasing water pollution and reducing water resources [2]. The effluents of various industries cause pollution of the water body and, as a result, contamination of the entire ecosystem [3]. On the other hand, a lack of water resources is a critical issue, especially in arid areas. Therefore, the use of new, stable, clean, inexpensive, and available methods and technologies for water purification is considered a fundamental issue [4,5].

The increase in organic pollutants in the environment is the result of technological progress in recent decades [6]. Organic pollutants are known as dangerous pollutants due to their toxicity, non-biodegradability, and usage in a vast number of areas including agriculture, the chemical industry, the dyeing and textile industry, urban runoff, and wastewater treatment plants. Organic pollutants, in addition to being mutagenic and carcinogenic, may have synergistic effects whose effects on human health and other organisms are not known [3,7,8]. These pollutants are generally difficult to decompose due to their strong chemical structure, threaten the global ecosystem by entering the food chain, and even at low concentrations endanger human health [9,10]. Organic pollutants in water resources include dyes, oil spills, aromatic substances, organic acids, and trace residues of pharmaceuticals, pesticides, herbicides, and fertilizers [11,12].

Environmental concerns regarding water treatment are not only limited to the removal of pollutants, but a sustainable environment through sustainable consumption is also important [13]. As a result, the use of approved methods and materials in circular engineering can reduce these concerns [14,15,16]. Since the use of organic or inorganic chemicals in the removal of pollutants may cause secondary pollution, biopolymers can be a good alternative for preparing various types of organic pollutant purifiers [17].

Considering the growth of industry, the increase in population, the lack of water resources, and the lack of sufficient knowledge about the effect of organic pollutants, it seems necessary to prevent them from entering the environmental cycle. This is generally achieved through porous materials. Although other methods such as coagulation [18], flocculation [19], and biological methods [20] have been employed for wastewater treatment, porous materials are less complicated and easier to use [4]. Porous materials are compounds that have pores with interactive sites in their structure. These pores allow molecules and ions to enter the porous structure. Porous composites have long been of interest, but the ability to control performance and create engineered porous materials has made them more attractive to researchers than in the past [4,21]. Engineered porous materials aid in water purification due to their pore size distribution, internal and external active sites, specific surface area, reusability, various functional groups and thus selectivity, stability in various conditions, electrostatic properties, and successful chemisorption through different techniques such as adsorption, membranes, degradation, or oxidation [22,23]. Porous materials are divided into two general categories based on their structure and chemical composition. The first generation includes zeolites, activated porous carbon, etc., and the second generation, which includes advanced and engineered porous materials, is divided into two categories: organometallic porous compounds (porous coordination polymers (PCPs) and metal–organic frameworks (MOFs)) and porous organic polymers (POPs) [21,24]. Although the first generation of porous materials have been studied as pollutant adsorbents for a long time and have shown a satisfactory ability to separate pollutants, the second generation of porous materials have received attention in recent years due to the possibility of designing adsorbents and improving their adsorption performance compared to the first generation [25]. Since most of the second-generation porous materials are prepared via laborious synthetic methods and lead to the emission of and increase in greenhouse gases, this prevents their use in practical applications [11]. Creating porous materials using biopolymers can reduce greenhouse gas emissions and prevent secondary pollution [26]. Also, these sustainable materials achieve the goals of green chemistry in the field of reducing hazardous substances in chemical reactions [23].

In this review article, the methods that are used for the treatment of pollutants are discussed first. Then, the latest progress in porous materials prepared from biopolymers that have been used in the removal of organic pollutants in recent years is investigated. The components of composite preparations, the reason for their use, the pollutant purification mechanism, and their performance in different conditions are surveyed, and the different composites are compared with each other. In the following, a vision of what work can be carried out regarding the removal of organic pollutants with the help of stable porous materials is mentioned.

## 2. The Fabrication of Porous Biopolymers

Biopolymers have drawbacks such as low porosity and vulnerability to harsh conditions like acidic and alkaline environments, as well as high temperatures. To overcome these drawbacks, various methods have been employed to create and develop porous biopolymer composites. These methods can be divided into three categories: nanoparticle composites, gel formations (hydrogel and aerogel), and alkaline pretreatment.

### 2.1. Composites with Nanoparticles

Nanoparticles can enhance porosity, boost strength and stability, and lower fabrication expenses. Nanoparticles used in the fabrication of porous biopolymers can be mineral or metallic. The dispersion of metal nanoparticles in the biopolymer structure improves and increases porosity. On the other hand, a porous magnetic composite can be obtained by selecting magnetic nanoparticles [27]. In addition to metal and magnetic nanoparticles, inorganic minerals such as hydroxyapatite can be used to increase porosity and strength, as well as increase the weight percentage of the designed composite. Along with all of the advantages that inorganic minerals have for the structure of composites, they reduce their pore size. In selective adsorption, determining the pore size is an outstanding advantage [28].

The performance of the composite can be specifically tailored by selecting the appropriate nanoparticle type. For instance, incorporating silver nanoparticles can enhance the composite’s antibacterial and antifungal characteristics [29]. Additionally, inorganic nanoparticles like calcium carbonate and hydroxyapatite are advantageous due to their abundance, affordability, non-toxic nature, degradability, and the availability of multi-verse adsorption sites, enhancing the composite’s strength [28,30].

### 2.2. Composites with Polymers

Furthermore, apart from complexing with nanoparticles, the formation of complexes with various polymers, including natural polymers, can be an effective method for enhancing porosity. For example, the addition of chitosan to quince seed gum results in the formation of a polyelectrolyte complex, which in turn changes the morphology and porosity of the final structure [31]. By incorporating polyethylene glycol or polyvinylpyrrolidone into chitosan, not only do they act as pore-generating agents but they also effectively boost the mechanical strength of the chitosan [14,32,33]. Foams and sponges can be stabilized using organic and inorganic compounds. CMC foam is stabilized through crosslinking with polyacrylamide and in the presence of divalent metal salts [34]. By utilizing a combination of various biopolymers, the effectiveness of sponge composites in eliminating pollutants is significantly improved. This can be attributed to the multitude of functional groups that are present, resulting in enhanced performance and efficiency in the removal of pollutants [35].

### 2.3. Composites with Metal Structures

Among the metal structures that improve biopolymer properties in combination with them, we can mention metal–organic frameworks (MOFs) and double-layer hydroxides (LDHs). LDHs, which possess a relative level of porosity, are useful as pollutant adsorbents. In combination with biopolymers, their porosity and surface area increase [36]. Furthermore, the composition of metal fillers in the biopolymer structure enhances their mechanical properties [37,38]. MOFs are crystalline species with high porosity that can improve the porosity of biopolymers [39]. Conversely, biopolymers occupy the pores within the porous structure of MOFs. Unlike the reduction in porosity, the performance of the final composite in absorbing pollutants improves due to the presence of many functional groups in the biopolymer. It is possible to reach the maximum porosity by changing the ratio of components in the composite [40,41].

### 2.4. Gel Formation (Hydrogel and Aerogel)

Bio-hydrogels are created through the process of physically or chemically crosslinking biopolymers in their chain structure. The preparation of bio-hydrogels requires the utilization of a biopolymer, an ionic crosslinker, or a covalent crosslinker [42,43]. These hydrogels are insoluble in water and have the unique ability to absorb water, are highly flexible, and are capable of deswelling. Due to these properties, porous bio-hydrogels can serve as effective green adsorbents for various types of pollutants. Combining bio-hydrogels with metal structures like MOFs and LDHs, nanoparticles, and synthetic hydrogels helps to improve their mechanical strength [44]. Hydrocolloids and low-crosslinked mucilage are types of hydrogel-forming polymers that exhibit unique properties when interacting with water. Their ability to form gels or viscous dispersions in aqueous environments is a key characteristic that sets them apart from other types of polymers [45]. The combination of hydrogels with minerals makes them thermally stable, although it reduces the specific surface area. Hydrogels can be prepared as beads or utilized in 3D printers to fabricate scaffolds [46,47].

Aerogels, unlike hydrogels which can be porous or non-porous, possess a three-dimensional structure and high surface area, obtained through freeze-drying to preserve their porous nature. Bio-aerogels, derived from biopolymers like gelatin and cellulose, exhibit easy gelation due to the presence of oxygenated functional groups like hydroxy and carboxyl groups as well as the presence of amines [48]. Utilizing epoxide-assisted gelation presents a flexible approach to fabricating nanocomposite aerogels [49]. By utilizing crosslinking agents such as epichlorohydrin in the manufacturing process of biopolymer composites, a desirable pre-gel is formed [50]. Another method for preparing aerogels is the carbonization of biopolymers and the formation of porous carbon before the lyophilizing process [51].

### 2.5. Biopolymer Pretreatment

An approach to creating porous biopolymers involves utilizing alkaline pretreatment. During this process, an alkaline substance like soda is employed to break the biopolymer chain, resulting in shorter polymer chains. Subsequently, through re-crosslinking, the biopolymer transforms into a porous material with a regulated pore size. Chitosan and pomelo peel are examples of biopolymers that have been processed into porous form using alkaline pretreatment [52,53].

Acetone has demonstrated its efficiency as a porous agent in preparing porous chitosan, contributing to the development of hydrophobic characteristics [54].

## 3. Wastewater Treatment Methods

### 3.1. Membrane Separation

**Figure 1:**
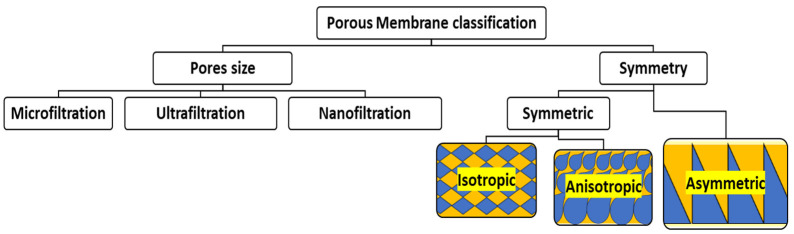
Porous membrane classification.

The membrane separation process has many benefits, including optimal energy consumption, simple operation, and the absence of chemical additives [55]. There are two types of structures for porous membranes. The first type is filtered based on the size of the pores, and it is divided into microfiltration, ultrafiltration, and nanofiltration according to the size of the pores. Ultrafiltration involves a pore size of 0.1 to 0.01 μm and is one of the most effective membrane separation processes for removing pollutants. The pore size for microfiltration involves 1–0.1 μm and nanofiltration involves 0.01–0.001 μm [56]. Another type of porous membrane is based on symmetry. Symmetrical porous membranes are divided into two groups, isotropic or anisotropic. Isotropic membranes exhibit a consistent structure and composition, typically categorized as microfilters according to their permeation rate. Additionally, they selectively separate contaminants. On the other hand, anisotropic membranes have a non-uniform composition with high permeability. They are used in pressure-driven membrane processes (reverse osmosis, ultrafiltration, and microfiltration). They consist of two layers, a selective thin layer that is backed up on a thick adsorbent layer. Asymmetric membranes, which are the most accessible membranes, consist of two layers: a thin upper layer with selective permeability and a porous layer below that in turn mechanically strengthens the membrane. The upper layer provides selective flux in comparison with the symmetric membrane by changing the preparation parameters (Figure 1) [23].

Since wastewater treatment is a running process, choosing the optimal membrane for wastewater treatment is difficult and depends on many variables. The nature of pollutants, membrane porosity, membrane manufacturing methods, and the separation process are among the effective variables in preparing and choosing the right membrane [23]. In addition to the mentioned variables, the high costs of preparing and restructuring the membrane should also be considered [9]. Membranes are classified based on their nature and are divided into two categories: synthetic membranes and bio-membranes. Synthetic membranes are primarily composed of synthetic organic polymers like polyamides and polyether sulfones, as well as mineral materials such as zeolites, ceramics, and metals. On the other hand, bio-membranes are derived from natural materials like biomass, chitosan, and cellulose. These natural materials can be combined with organic and inorganic substances such as carbon nanotubes, nano-fibers, transition metal oxides, and metal nanoparticles to enhance the membrane’s strength, and stability and even introduce photocatalytic properties. Utilizing natural polymers can be a viable solution to control the costs of membrane preparation [57].

### 3.2. Adsorption Treatment

**Figure 2:**
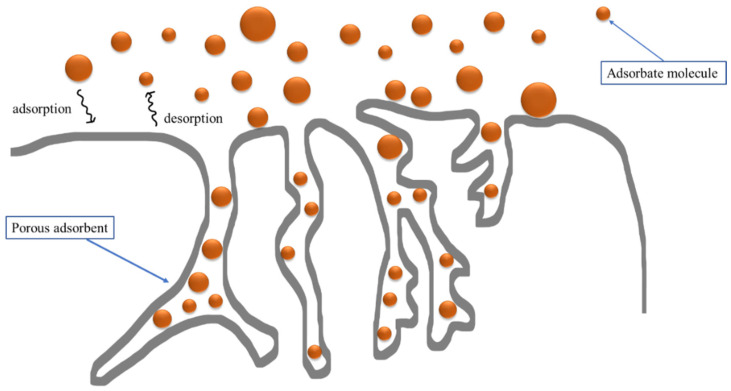
The concept of adsorption.

The adsorption process is one of the first methods of water purification, which is constantly increasing in importance and application [58]. Compared to other methods of pollution removal and wastewater treatment, adsorption is preferable due to its effectiveness and clean technique [59]. The adsorption process is actually the transfer of contaminating molecules and ions from the solution to the adsorbent solid network. Adsorption is performed on the surface of the adsorbent, and porosity is an important feature of adsorbents (Figure 2). Adsorbents are environmentally friendly due to their reusability, and if biomaterials are used for their preparation, they will be fully compliant with the principles of green chemistry and also inexpensive [60]. In addition to the mentioned cases, the possibility of designing the structure and performance of the adsorbent and appropriate efficiency are also among the merits of adsorbents. In fact, by designing an adsorbent using biomaterials, it is possible to reduce the costs of the adsorbent while having proper efficiency. Among the disadvantages of adsorbents are the dependence of their performance on pollutant concentration, the pH of the environment, and the solution temperature. It is difficult to prepare adsorbents that have their maximum performance in normal environmental conditions [23]. Other disadvantages of adsorbents include their lack of selectivity and the difficulty of separating them from the solution [9].

### 3.3. Degradation and Oxidation

**Figure 3:**
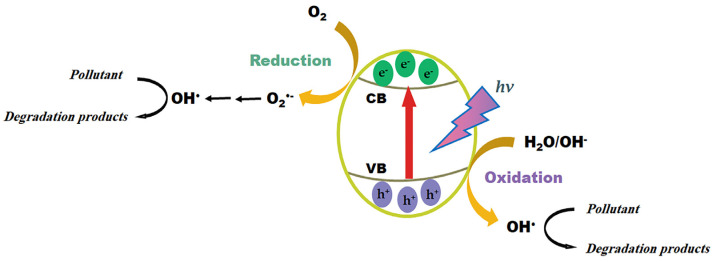
Schematic model of a photochemical reaction [6].

In the removal of organic pollutants through adsorption or using a membrane, there are still pollutants and this creates a problem in recovering the adsorbent or the membrane [61]. Degradation and oxidation are powerful methods in water treatment that belong to advanced oxidation processes [62]. In these methods, highly reactive oxygen species including hydroxyl radical and superoxide radical are prepared by a photocatalyst [63]. The oxidation method actually depends on a catalyst regardless of the presence of the oxidant. Generally, hydroxy radicals as active oxidizing agents oxidize pollutants [23]. Photocatalysts are water purifiers that are in the category of green chemistry. They are inexpensive and perform their catalytic activity in the presence of sunlight [64]. These compounds cause chemical changes in pollutants by adsorbing the pollutant and performing a redox reaction due to the presence of light. When the catalyst is exposed to light, electrons and holes are created. Photocatalysts are semiconducting compounds that perform either an oxidation reaction or a reduction reaction. The primary oxidant is the hydroxyl radical, which leads to the destruction of organic pollutants. Oxidation occurs in the valence band and degradation occurs in the conduction band. The transfer of electrons from the valence band to the conduction band causes the continuation of the photocatalytic process (Figure 3). If the band gap is large, only UV light can cause electron transfer between the VB and CB. The presence of elements doped with photocatalysts allows the band gap to be reduced and increases the number of radical species [6]. Metal centers and organic ligands can control their band gap and photocatalytic activity. Coordinating polymers reduces the chance of electron-hole recombination and helps to improve photocatalytic properties [24].

## 4. Organic Contaminates

### 4.1. Dyes

**Scheme 1:**
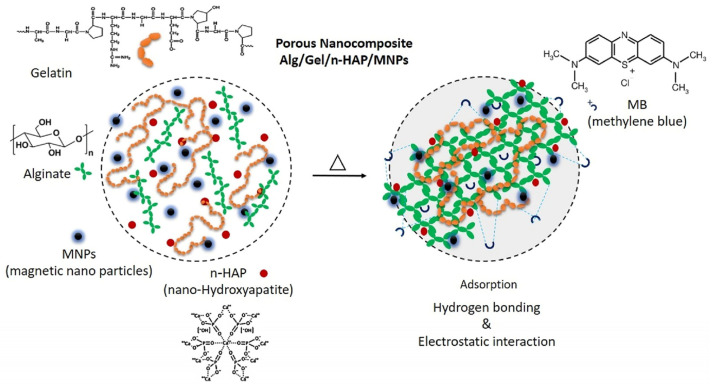
Schematic illustration of the synthesis of Alg/Gel/n-HAP/MNPs and the adsorption of dye by the prepared porous nanocomposites [28].

Wastewater from the textiles, plastic, and cosmetics industries contains anionic and cationic organic dyes which are very toxic, carcinogenic, and mutagenic, do not degrade in biological processes, and, on a great scale, spoil the appearance of water [65]. Thus, removing them from the wastewater and as a result removing them from the ecosystem is a vital matter. Inorganic coagulants that are often used cause sludge production and the effects of their use are still unclear. The use of bio-porous organic materials is a reliable method to remove organic dyes [23]. In Table 1, a summary of using porous biopolymers in the adsorption of dye can be seen. For example, a magnetic porous organic-inorganic adsorbent was prepared by Zhou et al. (2024) to adsorb methylene blue, a cationic phenothiazine dye (Scheme 1). Alginate and gelatin biopolymers as adsorbent organic parts are biodegradable materials with high adsorption capacity. The polymer network created by carboxyl groups in alginate and gelatin leads to the trapping of dye molecules and improves adsorption capacity. Hydroxyapatite as an adsorbent mineral part improves the physical, mechanical, and adsorption performance of synthesized porous nanocomposites [66]. Adding mineral additives to porous biopolymer composites, along with improving their hydrothermal stability, also reduces the costs of adsorbent synthesis. Based on the research results of Zhou et al., increasing the amount of hydroxyapatite by 30% by weight of alginate improves the porosity, compressive strength, and elastic modulus, but it reduces the pore size by about 45% and affects the swelling of the adsorbent. Also, dye adsorption studies showed that the highest q_e_ is at 60 °C and equal to 561.5 mg/g if the percentage of composite components based on the alginate weight is equal to 30% hydroxyapatite and 40% gelatin. The adsorption of methylene blue by nanocomposites is an endothermic process, and increasing the temperature activates the network structure of the composite [28].

Gelatin is a biopolymer with many functional groups such as amine, carboxyl, and hydroxyl groups, which makes it a good absorbent. A gelatin composite with other mineral nanofillers can also act as an efficient absorbent. In the study conducted by Elella et al. (2024), it was shown that montmorillonite nanofiller, in addition to increasing mechanical strength, improves its porosity and increases absorption capacity. The addition of 3% montmorillonite to a gelatin–acrylamide (AAM)–itaconic acid (IA) co-polymer hydrogel in the presence of the N, N′-Methylene-bis-acrylamide crosslinker leads to the preparation of a nanocomposite with suitable porosity, sufficient thermal stability, and an absorption capacity of 950 mg/g. Increasing the amount of montmorillonite nanofiller increases thermal stability, but by reducing the porosity of the gelatin co-polymer reduces its performance in removing the malachite green color [67].

Chitosan is also one of the abundant cellulosic biopolymers with a significant number of amine functional groups, which can be used as a dye absorbent with suitable porosity and durability as a composite with minerals such as montmorillonite, bentonite, kaolinite, zeolite, and hydroxyapatite (Hap). Billah et al. (2024) used a chitosan composite with zinc-doped hydroxyapatite to remove methyl orange dye. Investigations showed that the ratio of Zn^2+^@Hap/Cs between 30 and 50% W has the same and higher absorption rate than other weight ratios. A porous structure with a surface area of 57 m^2^/g and the presence of mineral filler improves the thermal stability of the composite. The maximum absorption capacity in optimal conditions is 453.15 mg/g. It is possible to exploit this composite on an industrial scale due to the lack of dependence on the exact amount of the mass ratio of the constituent materials, as well as their abundance and cheapness [68].

In addition to nanocomposites, organometallic frameworks, which are a subset of coordination polymers, can also benefit from biopolymer properties in combination with them. MOF-based adsorbents have a high surface area, low density, stability, and the possibility of post-modification. In addition to all the advantages of metal-organic frameworks as efficient adsorbents, they also face limitations due to the high costs of synthesis and recovery [69]. Therefore, combining them with biopolymers, in addition to being economical in the synthesis of these compounds, also increases their stability [70]. In the research conducted by Hegde et al. (2023) in the combination of keratin extracted from human hair (HA) with Ce-UiO-66 MOF, it was found that the presence of keratin with amino and hydroxy functional groups increases dye adsorption. Although in the results obtained from BET, we see a decrease in the surface area of Ce-UiO-66 MOF after combining with HA, which occurs due to the occupation of the empty spaces of Ce-UiO-66 MOF with keratin, the electrostatic interaction, hydrogen bonding, and π–π interaction not only compensate for the decrease in porosity but also improves the side of adsorption [40].

Due to the importance of sustainable consumption, biomass has been considered very important over the last decade. These materials can be used for energy production, carbon sequestration, and soil improvement in agriculture and have the potential to replace petroleum materials [71]. Biopolymers obtained from biomass are biocompatible, biodegradable, and available like other biopolymers [72]. Another advantage of them is their cheapness, abundance, and contribution to the sustainability cycle [73,74]. For example, polyhydroxyphenols can be obtained from tannin in an alkaline medium. Sadegh et al. (2022) extracted tannin from the skin of Zagros Mountain oak by using the alkaline method. The extracted tannin after polymerization with formaldehyde and magnetization (Fe_3_O_4_–OT) was able to simultaneously remove cationic malachite green and anionic sunset yellow dyes with high yield. Adsorption occurs through hydrogen bonding and electrostatic attraction. The results of the pH test showed that the adsorbent in the alkaline environment has more adsorption in the cationic dye and vice versa [75].

**Table 1:** Porous biopolymers in the adsorption of dye.

Adsorbent	Biopolymer	Other Components	Dye	q_e_ (mg/g)	Ref.
Grafted gelatin/MMT nano clay nanocomposites	Gelatin	Montmorillonite, Acrylamide (AAM)—itaconic acid (IA) n, n′-methylene-bis-acrylamide	Malachite green	950.5	[67]
Nanocomposites Alg/Gel/n-HAP/MNPs	Alginate and gelatin	Nano-hydroxyapatite and magnetic iron oxide nanoparticles (MNPs)	Methylene blue	561.5	[28]
Ce-UiO-66 MOF @Keratin composites	Keratin	Ce-UiO-66 MOF (1,4-benzene dicarboxylic, dimethylformamide, ammonium ceric nitrate)	Trypan blue	469.5	[40]
Nanocomposite Zn^2+^@HAP@CS	Chitosan	Zinc-doped hydroxyapatite	Methyl orange	453.15	[68]
Composite biopolymer sponge GO-coated PS	Gelatin and chitosan	Poly (vinyl alcohol) and graphene oxide	Congo red	129.53	[35]
			Rudamin B	99.53	
Cs/PEG composite membrane	Chitosan	Polyethylene glycol/TEOS	Methyl orange	74 mg/g	[32]
POP magnetic oak tannin gel (Fe_3_O_4_–OT)	Tannin gel	Fe_3_O_4_	Malachite green	49.00	[75]
			Sunset yellow	53.95	
CMC foams	Carboxymethyl cellulose	Poly (acrylic acid), CaCl_2_	Methylene blue	Not mentioned	[34]
Polyelectrolyte complexes CS-QSG	Chitosan and Quince seed gum	-	Methylene blue	30.88	[31]
			Rudamin B	15.16	
			Methyl orange	14	

Polymer sponges are known as new and cost-effective adsorbents due to their low density, high porosity, flexibility, and high endurance in different environmental conditions [76]. Hydrophilic sponges with hydroxy, carboxyl, and amine functional groups are prone to productivity issues in the textile and agricultural industries. Making and preparing these sponges with biopolymers reduces the biological concern of using this effective compound. Vo et al. (2022) prepared a polymer sponge from crosslinked polyvinyl alcohol, chitosan, and gelatin (PS) and used the composite of this polymer sponge with graphene oxide (CS) to adsorb anionic and cationic dyes. The prepared sponge composite is inexpensive, readily available, biocompatible and safe and has the potential for large-scale use. The protonation of hydroxy and amino groups and the deprotonation of carboxyl groups in the sponge adsorbent cause the electrostatic adsorption of pollutants. Composite polymer sponges with graphene oxide have a positive effect on dye adsorption and their functional groups interact with dyes. Also, the adsorption of Congo red is better than rhodamine B, which is probably due to the steric hindrance around the amine groups in rhodamine B. However, this sponge adsorbent can have good performance in adsorbing cationic and anionic dyes [35].

**Figure 4:**
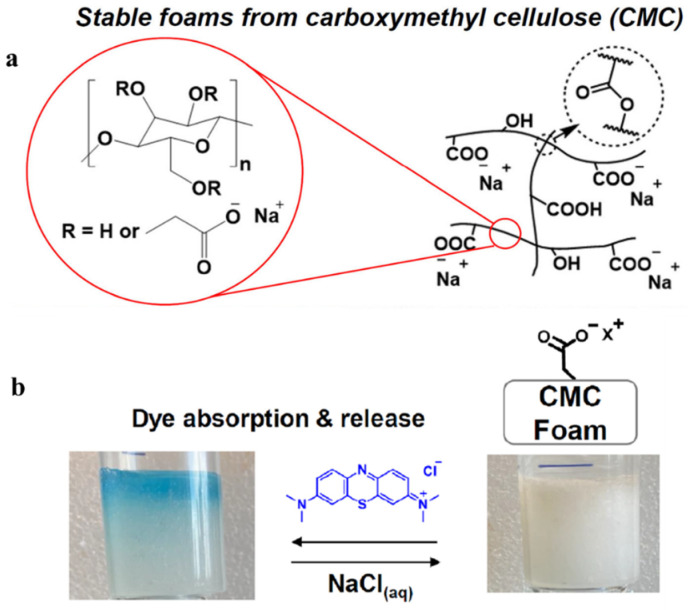
Stable foam structure from CMC (**a**) and schematic of the adsorption/desorption of methylene blue using CMC foams (**b**) [34].

Anionic bio polyelectrolytes can adsorb cationic dyes. To use biopolymers such as CMC, it is necessary to modify their structure to prevent their dissolution in water and to be able to be use them as a foam with suitable porosity to adsorb organic pollutants. Cui et al. (2023) prepared a reinforced foam from CMC and two ionic crosslinking agents (calcium chloride) and covalent (polyacrylic acid) (Figure 4a) and showed that the resulting foam exhibits insolubility in water, along with possessing suitable porosity and the capacity to adsorb methylene blue dye. Furthermore, it can reabsorb dyes within a sodium chloride saline environment (Figure 4b) [34].

**Figure 5:**
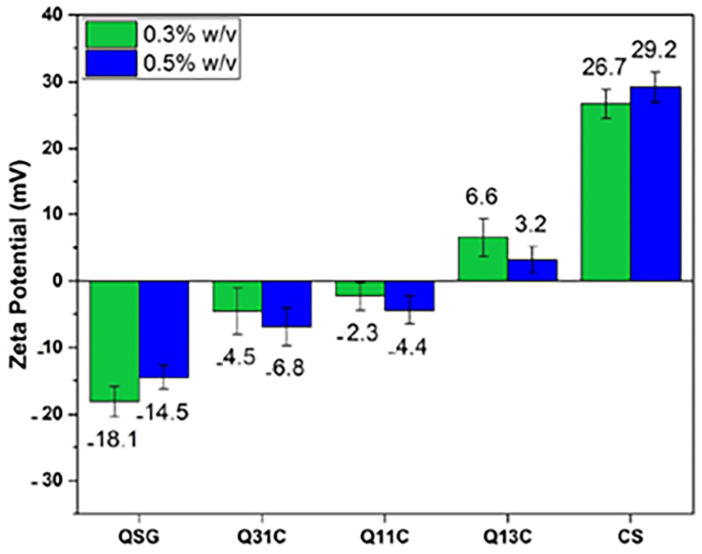
Zeta potential of QSG, CS, and PECs prepared at different concentrations and volume ratios [31].

Polyelectrolyte complexes are created from the electrostatic interaction between polyions, and due to the presence of two polyions with opposite charges in a complex, it is possible to adsorb several colors (anionic and cationic) in these compounds. Kaviani et al. (2023) prepared a polyelectrolyte bio-complex from chitosan (CS) as a polycation and Quince seed gum (QSG) as a polyanion and investigated its performance in adsorbing methylene blue, rhodamine B, and methyl orange. Investigations showed that electrostatic interactions between amine groups in chitosan and carboxylic acid in Quince seed gum are effective in zeta potential values, and whichever polyelectrolyte had a higher percentage was more effective in zeta potential value (Figure 5). In addition to electrostatic interaction, the porous polyelectrolyte consists of hydrogen bonding interactions as well as hydrophobic interactions. The SEM images show the porosity of the surface of the complex, which was the first porosity related to the ratio of 3 to 1 quince seed gum to chitosan with a concentration of 5% by weight/volume. Also, the removal percentage of cationic dyes in this complex was as expected and more than anionic and zwitterionic dyes [31].

**Scheme 2:**
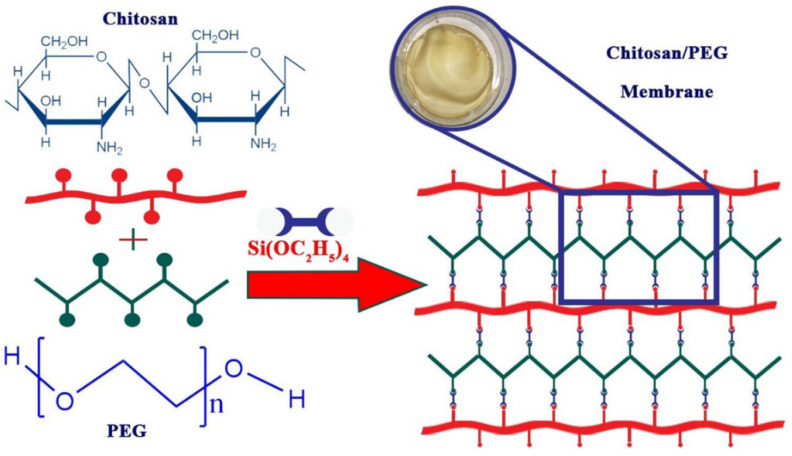
Schematic illustration of a chitosan/PEG membrane [32].

Another method of adsorbing colors from aqueous media is the use of porous membranes. Chitosan can be a good adsorbent for anionic dyes due to it having amino groups in acidic environments. However, since this biopolymer is fragile and does not have proper strength, it can be helpful to composite it with other polymers in preparing porous membranes. Ahmad et al. (2023) prepared a chitosan membrane composited with polyethylene glycol to adsorb methyl orange (Scheme 2). Polyethylene glycol, in addition to creating strength, is the cause of creating pores in the membrane structure. In this membrane, TEOS was used as a crosslinker, which, although it reduces the amino groups of chitosan, increases the viscosity of the membrane. SEM studies show that the methyl orange dye has been successfully adsorbed on the surface of the membrane. It is an isotropic membrane whose average pore size is 0.38 nm and is in the category of reverse osmosis membranes. As a result, only water molecules are permitted to permeate through the membrane, while other molecules are absorbed by the membrane. Adsorption takes place through hydrogen bonding between methyl orange sulfonic groups and hydroxy groups in the chitosan membrane, as well as electrostatic adsorption with the amino groups of chitosan. Among the advantages of this membrane, we can mention the adsorption capacity of 74 mg/g in a short time and at room temperature [32].

Reviews show that chitosan is the most widely used biopolymer in the preparation of dye absorbents in recent years, which could be related to the abundance of this biopolymer, its cheapness, and the presence of amino and hydroxy functional groups. Also, it seems that chitosan composites with inorganic fillers have a remarkable effect on performance compared to composites with other biopolymers and organic compounds. The second most widely used biopolymer in recent years is gelatin, which, in combination with inorganic materials, has a very good absorption capacity for cationic dyes. In general, the crosslinked composites of biopolymers show weaker performance compared to other composites.

**Figure 6:**
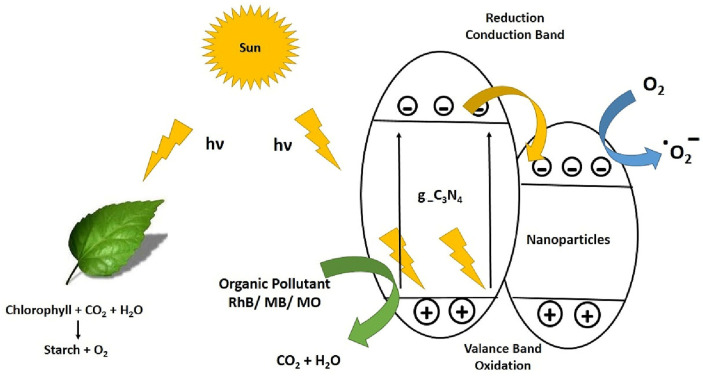
Photodegradation mechanism [53].

In addition to all of the advantages that adsorbents have in adsorbing colors, this method cannot decompose these pollutants, it can only stabilize them on a support. As a result, using catalysts that decompose them into simpler substances such as water and carbon dioxide, which are non-toxic, is a more suitable method [77,78]. Likewise, porous biopolymers that have photocatalytic effects can improve the performance of conventional photocatalysts. This idea, inspired by bulk heterojunction organic solar cells, by adding acceptors, leads to an adjustment of energy levels [79]. In Table 2, a summary of using porous biopolymers in the degradation of dye can be seen. For example, graphitic carbon nitride, with the ability to produce highly reactive oxygen species (ROS) forming electron–hole pairs when illuminated with light, is a well-known and widely used photocatalyst that has been used in many studies for the photocatalytic decomposition of dyes [80]. Porous chitosan could act as an amazing electron acceptor, avoiding the quick recombination of electron–hole pairs and, in this way, upgrading the generation of reactive oxygen species (Figure 6). Praseetha et al. (2023) showed that doping porous chitosan with graphite carbon nitride increases the surface area and also improves the photocatalytic process for the degradation of rhodamine B and methyl blue dyes. Also, investigations have shown that the synthesized porous photocatalyst acts faster in the presence of sunlight than in UV [53].

**Scheme 3:**
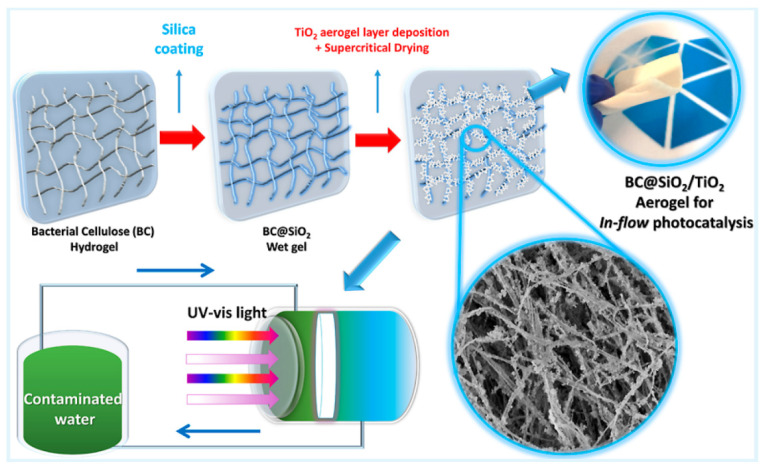
Schematic preparation and in-flow application of BC@SiO_2_−TiO_2_ [49].

The photocatalytic effects of transition metal oxide nanoparticles are of great interest due to their stability and excellent photocatalytic activity. However, the effects of metal oxide nanoparticles on the health of living organisms are still unknown [81], so it is better to immobilize them on a support in the form of a membrane, bead, or aerogel to facilitate their separation and operation. If the selected support is porous and biocompatible, it can increase the performance of nanoparticles and also reduce concerns about the entry of these particles into the ecosystem to some extent. Aerogels based on natural polymers are suitable substrates for metal nanoparticles, which, in addition to their high surface area, also have good mechanical stability in aqueous environments [82]. As a result, the combination of Aerogels and transition metal oxide can be considered as an excellent photocatalyst for removing organic pollutants, including dyes. Silva et al. (2023) prepared a hybrid aerogel photocatalyst of bacterial cellulose coated with silica and titanium oxide nanoparticles and investigated its application in the in-flow photocatalytic activity of methylene blue dye in a membrane photoreactor (Scheme 3). In this study, 100% color degradation was observed in 30 min under ultraviolet radiation. The existence of macropores and mesopores in the aerogel plays a significant role in enhancing the efficiency of photocatalytic pollutant degradation. This unique feature allows for better liquid diffusion within the material, ultimately leading to an increase in the catalytic area available for the degradation process. The use of a cellulose coating prevents the photocatalytic degradation of bacterial cellulose and increases the rate of color degradation but reduces the amount of titanium oxide nanoparticles in the aerogel. Therefore, it is important to determine the optimal coverage of cellulosic bacteria with silica [49]. Due to its photoactive crystalline phase and high resistance, TiO_2_ is considered a suitable metal oxide for the preparation of photocatalysts. Also, the presence of silica causes the formation of a pure anatase phase and reduces the disadvantages of TiO_2_ such as a broad band gap and the high recombination of electron–hole pairs [83].

Also, porous biopolymers can improve the catalytic oxidation performance of metal oxides. Manganese oxide is one of the transition metal oxides that is used in heterogeneous catalytic oxidation and has advantages such as multiple oxidation states, non-toxicity, and compatibility in difficult environmental conditions [84]. Among the disadvantages of oxidizing catalysts, we can mention the lack of ability to adsorb pollutants and the accumulation of their particles before catalytic oxidation. Porous biopolymers, due to them having many functional groups, can adsorb dye molecules into oxidative catalyst bulk. Also, porous biopolymers can be used as a support for the easier collection of metal oxide particles. Therefore, the formation of a biopolymer-support MnO_x_ catalyst effectively combines the advantages of both materials for simultaneous polymer capture and catalytic oxidative activation, ultimately ensuring the degradation of organic pollutants. Zheng et al. (2023) prepared a MnO_x_ oxidative catalyst together with pomelo peel (PP) biopolymer in a single step and used it to decompose methylene blue. The SEM images of this composite indicate that it has a porous biopolymer cavity-like structure, and manganese oxide is placed inside these cavities. Also, the EPR data showed that the manganese oxide–pomelo peels composite produces more reactive oxygen species, which leads to the strengthening of the oxidizing catalyst effect. XPS data also confirm the degradation of methylene blue to water and carbonate products [52].

Among other methods used to remove dyes, we can mention catalytic reduction. By splitting the azo bond in the dye, this method will lead to the formation of more stable products with less toxicity. The reduction of bonding requires the presence of electron-donating metal nanoparticles such as Ag, Zn, Ni, Cu, Fe, and Mn. In order to prevent the accumulation of the mentioned nanoparticles, it is necessary to stabilize them on a support [85]. Porous biopolymers, in addition to helping to adsorb contaminants in their porous structure and accelerating the reduction process, are cheap and biocompatible, and the prepared catalyst is in the field of green chemistry. Among the available biopolymers, alginate is of interest in the preparation of reduction catalysts due to the possibility of crosslinking with metals. Benali et al. (2023) prepared a zinc reduction catalyst on an alginate substrate with a copper crosslinking agent and used it to reduce organic dyes. The research results showed that the amount of copper crosslinking agent is effective in the amount of zinc nanoparticles doped to the composite, and the higher the amount of copper, the lower the remaining site content in alginate and the lower the amount of zinc metal nanoparticles. The mechanism of action in this catalyst is that the reducing agent of sodium borohydride creates a negative charge on the adsorbent surface and causes the adsorption of cationic dyes. Then, the pollutants penetrate into the alginate porous support. Next, metal nanoparticles transfer the necessary electrons for reduction from NaBH_4_ to dye molecules. Due to the electrostatic effects, methylene blue cationic dye elimination was more effective than that for other dyes. This catalyst has been used to reduce the colors of methylene blue, methyl orange, orange G, Congo red, and 4-nitrophenol [47]. Similarly, alginate composite beads with a copper crosslinking agent and silver nanoparticles were used as a reduction catalyst to remove methylene blue, 4-nitrophenol, methyl orange, orange G, and Congo Red pollutants. Silver nanoparticles are good at electron transfer and immobilizing them in an alginate polymer support can solve their accumulation and collection problems. Again, the ratio of crosslinking copper with silver nanoparticles is in reverse. Also, the best catalytic activity was observed for the reduction of methyl orange, followed by concord and 4-nitrophenol. The research results showed that the catalyst has no effect in reducing orange G [29].

**Table 2:** Porous biopolymer catalysts in the degradation of dyes.

Catalyst	Biopolymer	Other Components	Method	Dye	Constant Rate (t^−1^)	Ref
Porous chitosan-gC_3_N_4_ nanosheets	Chitosan	Graphitic carbon nitride	Photocatalytic degradation	Rudamin B	not mentioned (R% = 98)	[53]
BC@SiO_2_/TiO_2_ Hybrid Aerogel	Bacterial nanocellulose (Komagataeibacter xylinus bacteria)	TEOS and TiO_2_	Photocatalytic degradation	Methylene blue	0.1538 min^−1^	[49]
Cu-alginate (ZnNPs) hydrogel composite beads	Sodium Alginate	Zn (NO_3_)_2_ and Cu (NO_3_)_2_	Catalytic reduction	Methylene blue	0.798 min^−1^	[47]
				Methyl orange	0.456 min^−1^	
				Congo red	0.216 min^−1^	
				Orange G	0.252 min^−1^	
AgNPs@Cu@ Alginate aerogel Composite beads	Sodium Alginate	AgNO_3_ and Cu (NO_3_)_2_	Catalytic reduction	Methylene blue	0.0061 s^−1^	[29]
				Methyl orange	0.0131 s^−1^	
				Congo red	0.0016 s^−1^	
				Orange G	0	
Alg/XG/AgNPs/Dex/Ca nanocomposite	Alginate/Xanthum Gum/Dextran	AgNPs and CaCl_2_	Catalytic reduction	Methylene blue	0.31907 min^−1^	[86]
Nanocopper/ Chitosan aerogel biocomposite	Chitosan	CuSO_4_.5 H_2_O and extract of durian shell (DS)	Catalytic reduction	Methyl orange	0.163 min ^−1^	[87]
Metal-oxide catalysts MnO*_x_*-PP	Pomelo peels	Manganese oxide (MnO*_x_*)	Catalytic oxidation	Methylene blue	not mentioned (q_t_ = 50)	[52]

Silver nanoparticles are widely used as a reducing catalyst due to their significant electron transfer ability. In another study conducted by Shrivastava et al. (2024), a reducing hydrogel containing silver nanoparticles was prepared from alginate and dextran biopolymers along with xanthan gum. Alginate and dextran have the role of stabilizing silver particles due to the presence of general groups. On the other hand, xanthan gum increases viscosity and color adsorption and improves hydrogel performance. This superabsorbent hydrogel has the ability to reduce methylene blue [86]. Truong et al. (2024) used economical copper nanoparticles as a reducing catalyst in an aerogel obtained from chitosan. Amino functional groups are a good substrate for bonding with copper particles. What makes this aerogel stand out is the use of the extract of durian (Durio zibethinus) shell (DS) as a reducer to convert copper cations into copper nanoparticles. Investigations have shown that the presence of copper, contrary to the reduction of porosity in the aerogel, improves its catalytic performance in reducing methyl orange [87].

The presence of metal nanoparticles is essential for the preparation of reducing catalysts. Sodium alginate is the most widely used biopolymer for the preparation of dye-reduction catalysts in recent years. The ability to create crosslinks with metals and carry metal nanoparticles in the network structure has made this biopolymer a suitable composition for the preparation of reducing catalysts. In the photocatalytic degradation of dyes, biopolymers have a positive effect on the performance of organic and inorganic photocatalysts such as C_3_N_4_ and TiO_2_.

### 4.2. Trace Organic Matter

Pesticides are pollutants in the agricultural industry that cause the pollution of waterways and the creation of toxic algae [88]. Paraquat is an organic pesticide that acts quickly and non-selectively and destroys the green tissue of plants. This compound has fast and high solubility in water, and since it decomposes very slowly, it causes bioaccumulation in the ecosystem. Although its use has been banned in many countries, it is still used in some places. Recent research findings have provided further evidence of the potential link between this compound and Parkinson’s disease [89,90]. Adsorption technology has been declared as one of the most suitable methods for removing this compound. An alginate porous biopolymer was used in the preparation of 3D-printed scaffolds. Baigorria et al. (2023) prepared a nanocomposite hybrid from the combination of sodium alginate and a bentonite biopolymer and used it as a bio-ink in nanocomposite 3D printing. The process of 3D printing makes it possible to prepare the adsorber in the desired shape and reduces the number of the preparation steps for the nanocomposite. Bentonite is a natural nano adsorbent composed of montmorillonite, which is used to remove inorganic and organic pollutants. Sodium alginate is used as a porous support for bentonite mineral adsorbents. In addition to forming a suitable hydrogel in the scaffolds of the 3D-printed product, alginate is also a biodegradable polysaccharide. A hydrogel prepared with the optimal percentage of constituents was studied for the adsorption of paraquat pesticides in different conditions. Analytical studies have shown that the addition of 30% by weight of bentonite increases the consistency of the ink compared to lower amounts of bentonite and maintains its adhesiveness and spreadability. Also, the optimal amount of bentonite showed the highest water adsorption capacity in the hydrogel, which was equal to 223.01%, which is probably due to the arrangement of bentonite particles in the sodium alginate network and doubles the absorption capacity. Due to the high thermal stability of bentonite, increasing its amount increases the stability and thermal resistance of the hydrogel. Since bentonite has a partial negative charge, the adsorption of paraquat, which is a cationic pollutant, decreases in acidic environments. As a result, maximum adsorption is observed in neutral environments and electrostatic effects remove the paraquat pollutant. Isotherm studies have shown that the adsorption process occurs homogeneously on the active sites and the presence of mineral salts decreases adsorption efficiency due to the competitive effects of the salt cation with the pollutant [46].

**Scheme 4:**
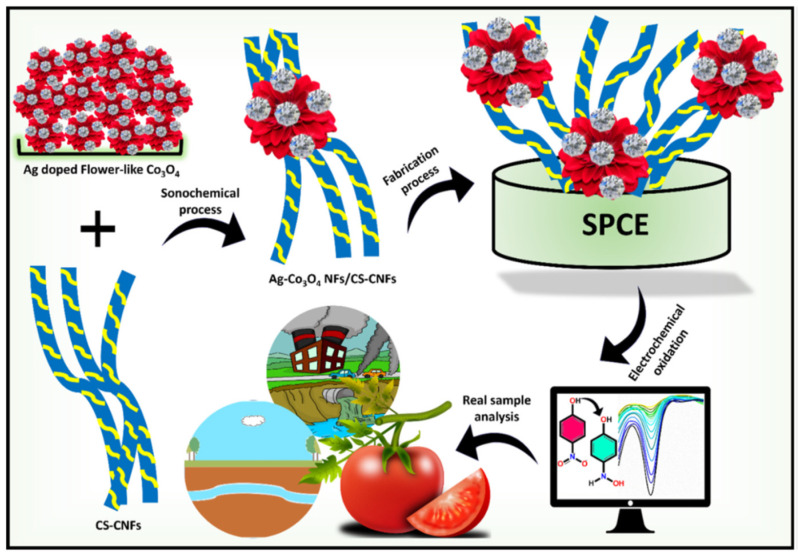
Schematic illustration of Ag–Co_3_O_4_ NFs/CS-CNFs/SPCE preparation for 4-NP detection [93].

4-Nitrophenol is one of the other polluting organic compounds in water. Even in small quantities, it poses a significant threat to both the ecosystem and human health. this combination changes the taste and smell of water, making it undrinkable. Since this pollutant is used in the plastics industry, the preparation of pesticides and insecticides, the preparation of paints, and the defense industry, it is abundantly observed in water sources. This phenolic compound is stable and very soluble. Hence, this pollutant is a priority for removing toxic materials [91,92]. Balram et al. (2022) used a porous nanocomposite prepared based on a chitosan biopolymer for the photocatalytic degradation and electrochemical investigation of 4-nitrophenol. To prepare this ternary nanocomposite, silver was anchored to Co_3_O_4_ with the help of UV radiation and attached to carbon nanofibers functionalized with chitosan through the sonochemical method. A screen-printed carbon electrode (SPCE) coated with a nanocomposite was used for electrochemical investigations. The use of two metals, cobalt and silver, to benefit from the properties of both, led to the creation of a bifunctional composite. Cobalt oxide is an antiferromagnet that has poor electrical conductivity. Anchored silver in cobalt oxide enhances the conductivity of the compound. Carbon nanofibers with surface activity increase electrochemical performance. In addition to strengthening the nanocomposite, chitosan strengthens the electrocatalytic properties due to it having many functional groups with the possibility of hydrogen bonding. Electrochemical investigations confirmed the selectivity of the prepared sensor against the desired pollutants (Scheme 4) [93].

Examining the recently prepared bio composite in the removal of trace organic matter confirms the evidence obtained in the removal of dyes. By adding some inorganic nanoparticles, the performance of alginate as an adsorbent increase significantly. Also, silver nanoparticles in combination with biopolymers are a suitable reducer to degrade dyes and have an effective function in reducing other nitrate organic compounds.

### 4.3. Organic Acids Pollution

**Scheme 5:**
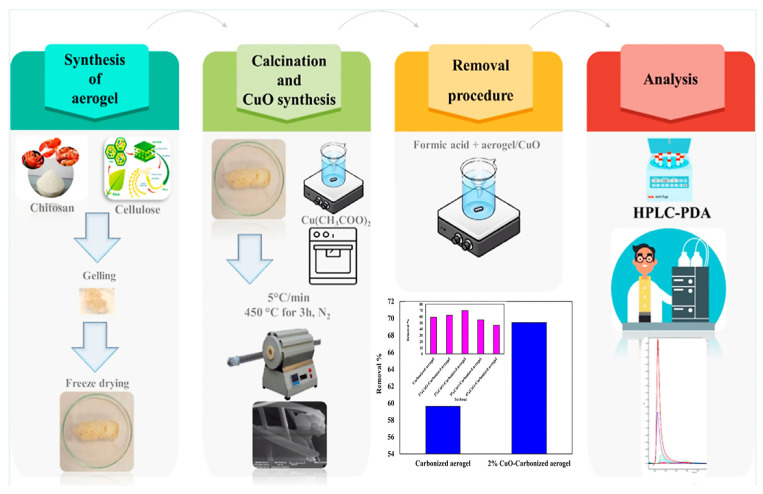
Schematic illustration of calcinated aerogel preparation and comparison of the efficacies of different CuO-modified aerogel sorbents in the removal of formic acid from water [50].

Organic acids are carbon compounds with acidic properties. Alcohols such as phenol and carboxylic acids are among the most widely used organic acids in industries [94]. These compounds are used as preservatives in agriculture and animal husbandry industries. For example, formic acid is used as an agent to eliminate salmonella bacteria in small animal feed. Petrochemical industries, pesticides, tanneries, and refineries are among the industries that produce organic acid pollutants, including formic acid [95]. Although formic acid does not have a harmful effect in the permitted amounts, but in case of repeated exposure to this substance, the tolerance level decreases and skin sensitivity occurs [96]. Amidi et al. (2023) prepared a porous aerogel from the pyrolysis of cellulose and chitosan and investigated the adsorption of formic acid. The results showed that the addition of copper acetate before the pyrolysis process improves the adsorbent performance and converts the added metal into copper oxide. The presence of metals activates adsorption sites by increasing electron exchange and facilitates ion exchange reactions. They also increase the physical and chemical resistance of the adsorbent. The performance of the adsorbent containing 2% copper oxide is 10% better than the adsorbent without it (Scheme 5). On the other hand, adding the amount of copper reduces the performance by occupying the pores of the adsorbent [50].

### 4.4. Organic Solvent and Oil Contaminate

Oil spills are unavoidable contaminations in the process of oil extraction in the sea that have harmful effects on the environment [97]. This pollutant is not only limited to the seas, but by spreading in the water due to water currents, it causes the pollution of beaches and marshes and affects the ecosystem [98,99]. There are different ways to clean the sea of oil materials. Among the traditional methods, we can mention burning on the spot and using canvases and skimmers [100]. New methods of cleaning are the membrane bio-reactors, the use of fillers and solidifiers, and the use of adsorbents [101]. Among newer methods, adsorbents are of interest due to their simplicity of use and there being no secondary pollution or damage to the environment [102]. A suitable adsorbent in this process must be hydrophobic and have a high oil adsorption capacity. Being cheap and biocompatible are other features that a suitable adsorbent must have. Bio aerogels can be a suitable adsorbent for this purpose due to their high porosity, low density, and naturalness. Maroulas et al. (2023) succeeded in preparing an aerogel that is completely hydrophobic, porous, and biocompatible and used biopolymer chitosan, polyvinyl alcohol, and reduced graphene oxide modified with methyl trichlorosilane. In this aerogel, chitosan was used to increase the electrostatic and van der Waals adsorption of oil particles. Graphene oxide has an aromatic hydrophobic and hydroxyl hydrophobic part, which makes it connect with other polymers and provides the possibility of forming a three-dimensional structure, which also prevents its dispersion. The use of reduced graphene oxide in this research causes graphene to bond with amino functional groups in chitosan, which is responsible for the adsorption process and gives a more suitable structure for the adsorption of oily particles. Silane is used to make the surface of the aerogel hydrophobic and polyvinyl alcohol increases the mechanical properties of the composition. While graphene oxide shows better mechanical properties than reduced graphene oxide, reduced graphene oxide has more BET and higher adsorption power. The adsorption capacity of the synthesized hydrophobic aerogel is about 27 g/g and can be reused up to five times [101].

Similarly, another aerogel was prepared from graphene oxide, lignin, and sodium alginate, and its hydrophobic properties were enhanced by trimethoxymethylsilane. The presence of many oxygen groups and hydrogen bonds in the lignin and sodium alginate causes the formation of a three-dimensional structure in the aerogel. In addition to its hydrophobic properties, lignin reduces graphene oxide and disperses reduced graphene oxide. This aerogel shows the properties of superhydrophobicity. The prepared microstructure aerogel was used to separate n-hexane, dichloromethane, and chloroform [103].

**Figure 7:**
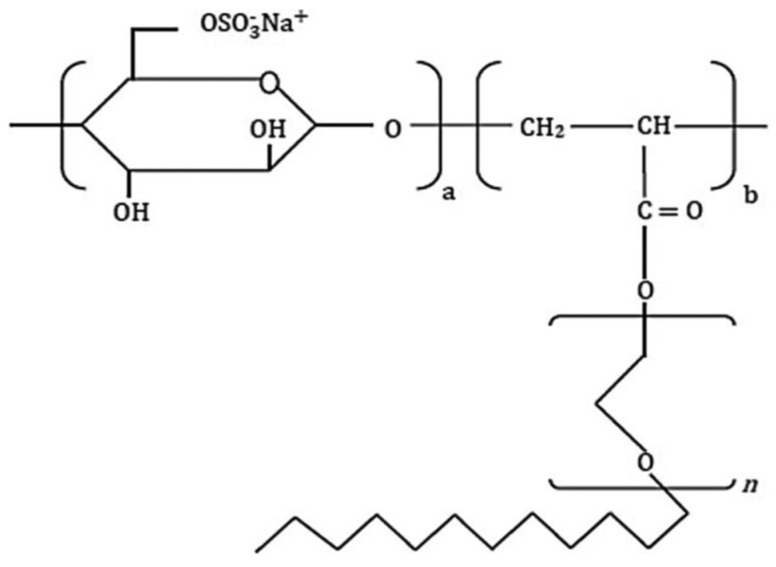
Molecular structure of novel hydrophobically modified sodium cellulose sulfate, DPEA-Cell-OSO^−3^ [104].

In another study, a hydrophobic cellulose surfactant was investigated for oil adsorption. In this research, sodium cellulose sulfate(a) was used as an inorganic ester. Placing hydrophobic polyethoxy functional groups such as dodecyl poly (ethylene oxide) acrylate(b) in the body of this cellulose derivative (Figure 7) allows its use in aqueous environments. Investigations have shown that this surfactant can be used in conditions similar to water environments in terms of temperature and salinity [104].

**Scheme 6:**
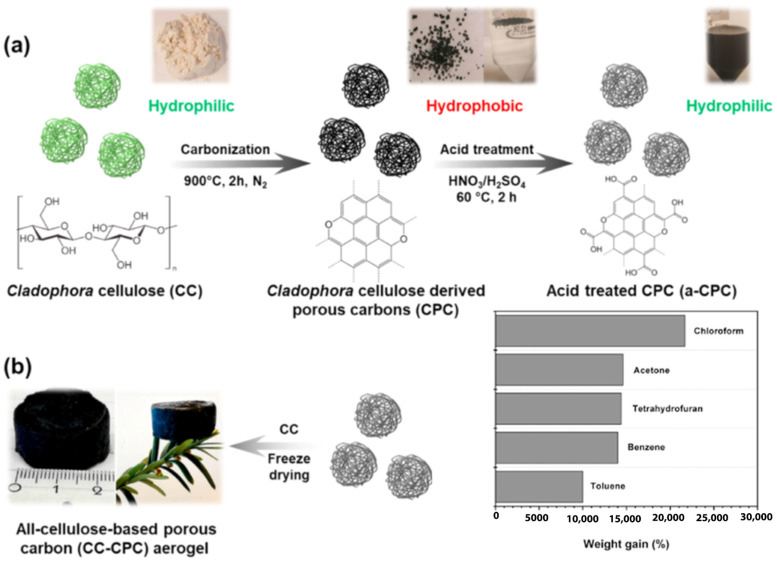
Schematic illustration of (**a**) the preparation of Cladophora cellulose-derived porous carbon (CPC) and acid-treated CPC (a-CPC) from Cladophora cellulose (CC) and (**b**) the formation of an all-cellulose-based porous carbon (CC-CPC) aerogel and the adsorption capacities of the aerogel for various organic liquids [51].

Organic solvents such as chloroform, acetone, tetrahydrofuran, benzene, and toluene are used in the manufacture of pharmaceutical products, shoes, paint, varnish, and glue [105,106]. Many organic solvents are classified as toxic or carcinogenic. They can cause water and ecosystem pollution [107]. Cellulose-based aerogels can be good candidates for removing these types of pollutants since they can adsorb more than 200 times their weight. Kong et al. (2022) prepared an aerogel from porous carbon obtained from Cladophora cellulose and formed a composite with Cladophora cellulose and prepared the desired aerogel during the process of freezing and freeze-drying. The results showed the excellent adsorption of this aerogel in the mentioned organic solvents, especially chloroform (Scheme 6) [51].

The use of biopolymers as adsorbents of oil and organic solvents, despite being economical and available, has the problem of dissolution in water and hydrophobicity. Therefore, modifying their structure with other compounds to increase hydrophobicity is inevitable. One of the methods of modifying the structure to make biopolymers hydrophobic is to add silane. Another method is to convert the soluble biopolymer into a polymer with a hydrophobic chain or to use carbonization methods. A summary of the discussed oil and solvent adsorbents is shown in Table 3.

### 4.5. Pharmaceutical Contamination

Although drugs have many benefits and restore human health, improper disposal of expired and unused drugs causes them to enter waterways, pollute the environment, and affect the growth of aquatic plants and bacteria [108]. The excretion of unmetabolized drugs and narcotics from the body is another source of contamination of wastewater with pharmaceutical pollutants [13]. Also, the entry of pharmaceuticals into the drinking water supply, coupled with frequent exposure to these substances, can enhance the body’s resistance to drug effects during the progression of illness [109]. Mutagenicity, bioaccumulation, and biological activity are other effects of contamination of water sources containing drugs. Also, due to their combination with chlorine, drugs can cause serious diseases such as respiratory diseases and mucous membrane irritation. In recent years, with the increase in the use of drugs due to the coronavirus disease, the amount of pharmaceutical pollutants in wastewater has grown significantly, and the issue of removing these pollutants has become more important than before [13]. Among the porous biopolymers that can be used to remove pollutants such as drugs is starch, which is the second most abundant biopolymer on the surface of the Earth. However, biopolymers generally have limitations due to their lack of strength and dissolution in water. One of the methods that solves these limitations to a suitable extent is the use of fillers such as layered double hydroxide (LDH) [38]. LDH has good performance in water purification processes, but its combination with organic materials increases the number of functional groups and porosity, thus increasing their performance. For example, Bansal et al. (2023) prepared an LDH of iron and nickel and different amounts of starch and investigated its performance in the removal of piroxicam. The results showed that adding starch causes more porosity, and doubling the amount of starch improves the maximum drug adsorption from 2531 to 2840 mg/g. Also, structural studies have shown that the addition of starch does not change the crystalline form of LDH [37].

**Figure 8:**
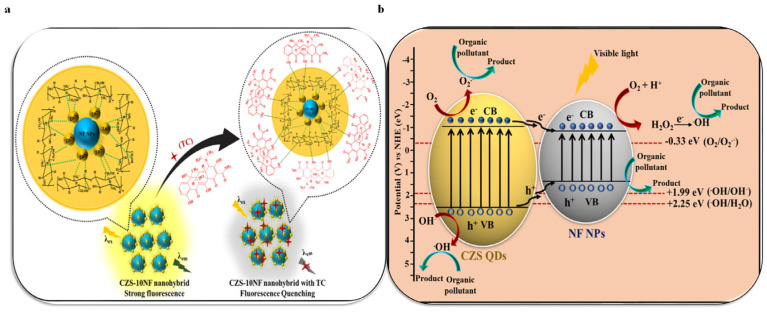
(**a**) Plausible mechanism for the fluorescence quenching of a CZS_10NF nanohybrid by TC. (**b**) Plausible mechanism for photocatalytic degradation of pharmaceutical drugs over a CZS_10NF nanohybrid [111].

The antibiotic tetracycline is an additive in animal feed. It accelerates the growth of animals and has antibacterial properties, and even its topical ointments are widely used. Its removal from the environment is a challenge due to the incomplete metabolism of this drug in the body of living organisms [110]. Kaur et al. (2024) designed a fluorescent sensor as well as a photocatalyst for the degradation of tetracycline (TC) and minocycline (MC) by capping chitosan to ZnS QDs and then magnetizing it with NiFe_2_O_4_ nanoparticles (NF NPs). Investigations showed that the fluorescence intensity decreases with increasing amounts of NF NPs, which shows that chitosan capped with ZnS QDs has a fluorescent nature. The fluorescent sensor has good detection power at a concentration below 20 μM, and its detection limit is at least 0.3 μM, which makes it a powerful sensor (Figure 8a). Also, this compound is an effective photocatalyst, and results have shown that the presence of NF NPs improved performance. It becomes photocatalytic because the energy level of the conduction band (CB) in NF NPs is lower and the energy level of the valence band (VB) is higher, and by reducing the required potential, this causes the transfer of electrons and holes to these nanoparticles (Figure 8b) [111].

**Scheme 7:**
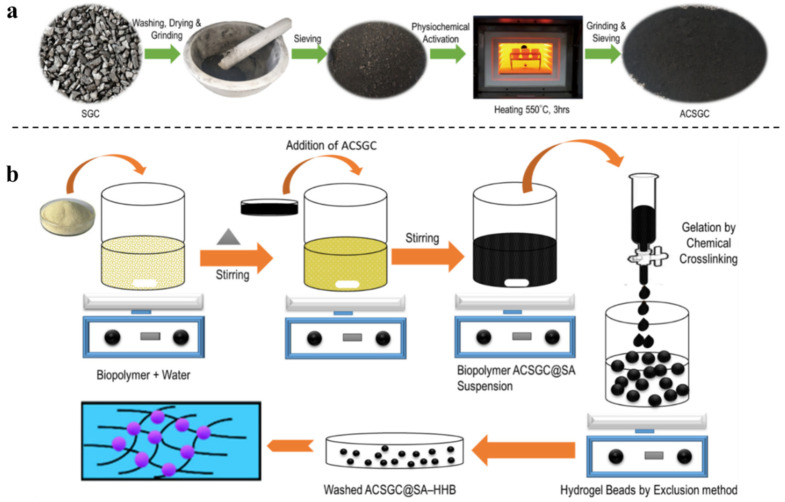
(**a**) Schematic for the preparation of AC-SGC activated carbon (AC) from spent granular carbon (SGC). (**b**). Schematic for the preparation of AC-SGC@SA-HHB [17].

Granular activated carbon (GAC) is an attractive adsorbent for removing various pollutants. This adsorbent with high porosity has good efficiency in adsorbing all kinds of organic and inorganic pollutants. But its recovery leads to the formation of activated carbon powder, which makes its performance in removing pollutants face the problem of removing the adsorbent and causing secondary pollution. Using activated carbon powders obtained from recycled activated carbon granules in the form of composites with biopolymers can solve this problem. The three-dimensional network of biopolymer hydrogels can be a suitable matrix for trapping activated carbon powder particles. These composites can be recovered and reused. Dever et al. (2023) prepared a hydrogel for the adsorption of the antibiotic tetracycline. They prepared beads using activated spent granular carbon (SGC) and sodium alginate and showed that this hydrogel could be recovered up to ten times. Also, the adsorption rate of tetracycline in the presence of this adsorbent was estimated to be 146.29 mg/g. Since this hydrogel is made from biomass and a biopolymer, it also follows sustainable consumption policies. Another prominent advantage of this hydrogel is the ability to recover up to 10 times (Scheme 7) [17].

Due to the presence of many functional groups such as hydroxy and amino, biopolymers can help in the adsorption of various pollutants, including drugs. In order to investigate this issue, Shahrin et al. (2023) used chitosan obtained from mud-crab (Scylla serrata) as an adsorbent for rifampicin (RIF), streptomycin (STM), and ibuprofen (IBU) and compared the performance of the resulting chitosan in adsorbing these three drugs. The maximum adsorption capacity of RIF, STM, and IBU was 66.91 mg/g, 11.00 mg/g, and 24.21 mg/g, respectively, which was higher than the other biopolymers obtained from agricultural waste, but compared to adsorbents containing activated carbon, which is a conventional adsorbent, the adsorption capacity was lower. The adsorption of pollutants in a neutral environment rejects electrostatic adsorption, so adsorption is carried out through hydrogen bonding on the surface of chitosan [112].

The unique properties of MOFs such as their high surface area, suitable porosity, and the preparation of engineered structure can also be used in drug adsorption. MOFs containing zirconium such as UiO-66-NH_2_, which has good physical and chemical stability, can be suitable adsorbents for various drugs. On the other hand, by modifying these compounds with the help of biopolymers, the problems related to their collection and recycling can be solved and their adsorption power can also be increased. Using gelatin, chitosan, and UiO-66-NH_2_, Kim et al. (2024) prepared an aerogel to adsorb ibuprofen and naproxen. The functional groups in gelatin improve adsorption, and the presence of chitosan increases the strength of the aerogel. Also, crosslinking chitosan and gelatin in the presence of UiO-66-NH_2_ strengthens the physical properties of the composite. The results of the investigations show that the presence of MOFs significantly improves the performance of the aerogel in removing the mentioned drugs [113].

A summary of the composites discussed in this section can be seen in Table 4. It seems that the use of LDHs has an effective role in improving the performance of biocomposites in removing drugs. Also, chitosan synthesized from mud-crab (Scylla serrata) shells shows better performance in removing ibuprofen compared to chitosan composited with gelatin and a metal–organic framework.

### 4.6. Natural Organic Matter (NOM)

**Figure 9:**
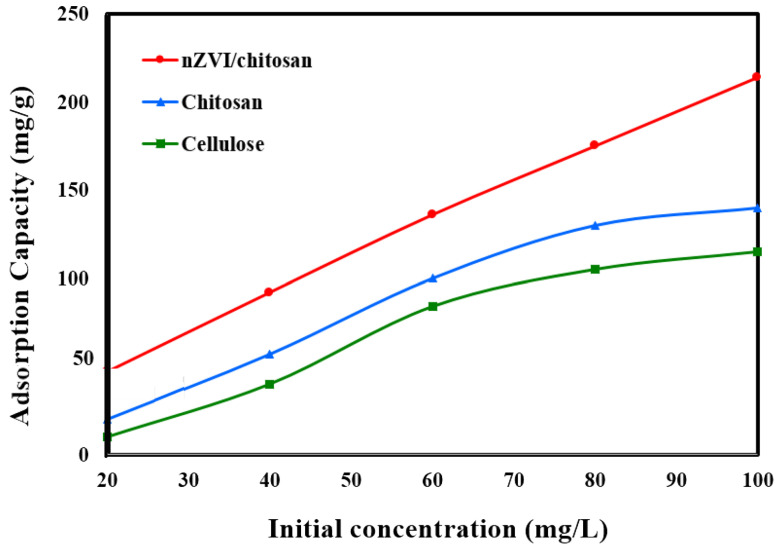
HA uptake into cellulose, chitosan, and nZVI/chitosan (pH 5.5, T = room temperature, t = 27 min, composite dose = 0.01 mg/L) [114].

Natural organic substances that include humic acid, fulvic acid, polysaccharides, proteins, and amino acids are among the influencing factors in water quality. Although humic acid is a useful compound for increasing soil fertility, it turns into strong carcinogens such as trihalomethanes in the chlorination process during water treatment. NOM also changes the appearance and taste of water. Also, this substance is the cause of goiter disease. Pourbaghaei et al. (2022) prepared a composite for the simultaneous removal of humic acid and nitrate with the help of chitosan and zero-valent iron and compared its performance with chitosan and cellulose (Figure 9). In order to benefit from the extraordinary properties of zero-valent iron nanoparticles, such as adsorption capacity, high surface area, and magnetic properties, it is necessary to cover them with biopolymers to prevent their accumulation and decrease in performance. Chitosan is one of the most widely used biopolymers, with it being abundant and available, non-toxic, and affordable. Also, many functional groups of this composition create suitable adsorbents. The as-prepared adsorbent has a core-shell structure, and the iron particles are well dispersed in the chitosan biopolymer. The selective adsorbent can remove 90% of nitrate and 98% of humic acid [114].

Kahloul et al. (2023) prepared a hydrophilic ultrafilter membrane for humic acid removal using cellulose acetate and Keggin polyoxometalate. Cellulose acetate alone has low mechanical resistance and is sensitive to temperature. In addition to increasing the mechanical-thermal resistance of the membrane, the addition of polyoxometalate keggin increases the porosity and flux of the membrane and its hydrophilic properties. Polyoxometalates are biocompatible and non-toxic, and along with other bio compounds, they can be a suitable compound for removing pollutants. The addition of polyoxometalate up to 15% improves membrane properties and has good performance in removing humic acid. Another advantage of this membrane is that humic acid adsorption does not depend on the pH of the environment; however, permeate flux retention is dependent on pH and decreases in an alkaline environment [56].

Diverse methods are employed by the biocomposites discussed in the text to eradicate pollutants. Investigating the mechanism behind porous biocomposites aids in enhancing our comprehension of their efficiency in eliminating pollutants and in the preparation of analogous composites that exhibit similar effectiveness. Table 5 presents a concise outline of the methods and mechanisms utilized for the elimination of contaminants.

## 5. Perspectives and Outlook

Organic pollutants, known for their detrimental effects on water quality, have the potential to induce various diseases and health complications, some of which have been identified thus far. Employing chemical substances in the creation of adsorbents and catalysts for the elimination of pollutants can result in the risk of secondary contamination. A more dependable approach in the treatment of wastewater involves the utilization of biopolymers to remove pollutants. The adoption of biopolymers as a means to eliminate pollutants offers a more sustainable and environmentally friendly solution compared to the use of chemical materials. By opting for biopolymers in wastewater treatment processes, the potential for secondary contamination can be significantly reduced, leading to a more effective and safer method of pollutant removal. Biopolymers have the potential to play a significant role in research aimed at eliminating organic pollutants, aligning with scientists’ wish for green and environmentally friendly chemistry. According to what has been mentioned about the properties and efficiency of biopolymers and observed in recent experiments, it seems that, in the future porous, biopolymer composites will play a crucial role in the future of environmental remediation. Biopolymers, despite their numerous advantages, are faced with certain weaknesses that hinder their widespread application. These limitations play a significant role in preparing and using them in various applications. By addressing these weaknesses and finding ways to overcome them, the potential of biopolymers to be utilized on a large scale can be maximized. One weakness factor to consider is insufficient strength, which can be significantly enhanced through the incorporation of mineral fillers. However, this enhancement comes at the cost of reduced porosity in the biopolymer. Another aspect to address is the requirement for lyophilization to create an aerogel porous biopolymer. The next issue is the lower efficacy of biopolymers compared to their synthetic counterparts in the removal of pollutants. The inherent solubility of biopolymers in water necessitates the utilization of supplementary techniques to fabricate composites that are insoluble in water. The extraction of biopolymers from biomass is a process that fully complies with the principles of circular engineering and green chemistry. However, the extraction and purification procedures associated with this process are known to be time-consuming and expensive. Furthermore, the final product’s yield is frequently suboptimal, posing a challenge to the extraction process.

A potential focus for future research lies in the synthesis of biopolymer composites with covalent organic frameworks. By compositing biopolymers with covalent organic frameworks, researchers can delve into the synergistic effects and unique characteristics that arise from this combination. This line of inquiry may lead to the development of advanced materials with enhanced functionalities, paving the way for innovative solutions in various fields such as medicine, energy, and environmental science. Covalent organic frameworks (COFs) exhibit remarkable characteristics such as high absorption capacity and lightweight, porous structures. These unique properties make them highly desirable for various applications, particularly in the field of wastewater treatment. It is expected that by compositing COFs with biopolymers, the process of separating COFs after treatment will become more efficient and dependable. The preparation of COFs from biopolymers also has the potential to revolutionize the field of COFs and facilitate their application in extensive industrial settings. By employing biopolymers as a starting material, the resulting bio-COFs will exhibit enhanced porosity and strength compared to conventional porous biopolymers. The utilization of biopolymers as a component in COF preparation represents a significant step forward in the quest for more efficient and robust COFs.

Porous biopolymer composites possess the capacity to absorb various types of medication, making them suitable for the creation of pharmaceutical complexes. Biopolymers that are biocompatible with living organisms and can be utilized in the formulation of these complexes. The pollution issue, which is partly caused by the presence of pesticides and herbicides in agriculture, can be addressed through the development of porous biopolymer composites. These composites can be created by incorporating fillers and micronutrient metals and can be subsequently employed in chemical fertilizers and soil enhancers. By implementing these measures, not only can agricultural conditions enhanced but the contamination of waterways and groundwater by toxic substances can also be effectively averted.

## 6. Conclusions

In this review, biopolymers as porous materials are effective in removing organic pollutants such as dyes in the wastewater generated by the textile industry, pesticides, insecticides, and chemical fertilizers used in the agricultural industries, the residues of expired or unmetabolized drugs in human sewage, oil pollution in the sea, and wastewater from the petrochemical industry were investigated. Organic pollutants are very toxic, carcinogenic, mutagenic, and extremely stable and in high concentrations cause changes to the color, smell, and taste of water. These pollutants are not removed in the usual water purification process and because of their high solubility, they spread quickly in the ecosystem. Due to the importance of sustainable consumption, biopolymers are a good candidate for replacement with other synthetic composites to remove pollutants. In addition to their porosity, biopolymers are biodegradable, biocompatible, non-toxic, readily available, and abundant. In addition to the mentioned advantages, these compounds, by having many diverse functional groups, provide the possibility of being composited with other compounds as well as removing pollutants more easily. Being obtainable from biomass is another prominent feature of biopolymers, which is also in line with green chemistry. Along with the many advantages mentioned for biopolymers, disadvantages such as low physical and chemical resistance, dissolution in water, and lower efficiency compared to synthetic porous composites can be mentioned. To solve this problem, combining biopolymers with other compounds such as metal nanoparticles, activated carbon, silicate, inorganic porous materials, and metal-organic frameworks and also crosslinking biopolymers with other polymers can be carried out. Composites obtained from porous biopolymers can be used in the form of adsorbent powder, membranes, aerogel, hydrogels, or photocatalysts. Composites obtained from intrinsic biopolymers have better performance. Investigations have shown that the addition of intermediate metals in the form of organic metal frameworks, zero-valent metal nanoparticles, and metal oxides improves the performance of porous composites in color degradation. Also, inorganic compounds such as hydroxyapatite and Montmorillonite in biocomposites will cause better absorption than metals and organic materials. The removal of organic solvents and petroleum substances is associated with the challenge of making biocomposites hydrophobic, which is generally achieved through the use of silica-containing compounds. The removal of medicinal substances by biopolymers depends on the presence of metals or traditional adsorbents such as activated carbon, but it is possible to prepare biopolymers with acceptable performance in removing medicinal pollutants.

## Figures and Tables

**Table 3:** Porous biopolymers in the adsorption of oil and organic solvents.

Adsorbent	Biopolymer	Other Components	Oil and Solvents	Adsorption Capacity	Ref.
CS/PVA/rGO /Graphene-based aerogels	Chitosan	Reduced graphene oxide, PVA, and methyltrichlorosilane	Diesel oil	27 g/g	[101]
T-SA/lignin/rGO-MTMS aerogel membrane	Lignin and sodium alginate	Reduced graphene oxide and trimethoxymethylsilane	n-hexane	11.8 g/g	[103]
			Dichloromethane	13.9 g/g	
			Chloroform	14 g/g	
			Isooctane	11 g/g	
			Pump oil	8 g/g	
			Xylene	5.5 g/g	
DPEA-Cell-OSO^−3^ polymer	Sodium Cellulose Sulfate	Ethylene ixide, acrylic acid, and dodecene	Oil	Not mentioned	[104]
Cladophora cellulose-derived porous carbons (CPC)	Cladophora cellulose (CC)	-	Chloroform, acetone, tetrahydrofuran, benzene, and toluene	100–217 (times own weight of sorbent)	[51]

**Table 4:** Porous biopolymers in the adsorption of drugs.

Adsorbent	Biopolymer	Other Components	Drug	q_e_ (mg/g)	Ref.
Starch-NiFe LDH (S/NiFe-LDH) composite	Corn starch	Fe (NO_3_)_3_.9H_2_O, Ni (NO_3_)_2_.6H_2_O	Piroxicam-20	1500.2	[37]
AC-SGC@SA–HHB composite hydrogel adsorbent	Sodium alginate	Activated carbon (AC) from spent granular carbon (SGC),	Tetracycline	22.6055	[17]
Chitosan derived from mud-crab (Scylla serrata) shell adsorbent	Chitosan (mud crab)	-	Rifampicin,	10.57	[112]
			Streptomycin	7.512	
			Ibuprofen	7.169	
gelatin–chitosan–MOF hybrid aerogels (CGC–MOF)	Gelatin Chitosan	UiO-66–NH2 (ZrCl_4_. 2-aminoterephthalic acid)	Ibuprofen	1.1	[113]
			Naproxen	1.4	
Chitosan-capped ZnS QDs/NiFe_2_O_4_	Chitosan	ZnS QDs (zinc nitrate and sodium sulfide) and NiFe_2_O_4_ (nickel nitrate and ferric nitrate)	Tetracycline	Rate constant * (k × 10^−3^ min ^−1^) = 21.7	[111]
			Minocycline	Rate constant * (k × 10^−3^ min ^−1^) = 28.7	

* Drug removal based on degradation.

**Table 5:** Remediation mechanisms.

Porous Composite	Method	Mechanism	Ref.
Starch-NiFe LDH (S/NiFe-LDH) composite	Adsorption	Physio-sorption process	[37]
Ce-uio-66 MOF @Keratin composites		Hydrogen bonding, π–π interaction, electrostatic interaction, and pore filling	[40]
Sodium alginate–bentonite clay (SA-B) nanocomposite hydrogels		Exothermic adsorption and electrostatic interactions	[46]
Cuo-cellulose and chitosan (CS/CE) aerogel		Exothermic, entropy-reduction, chemisorption, and single layer	[50]
Grafted gelatin/MMT nano clay nanocomposites		Electrostatic forces and coordination bonding (metal ions of MMT nano clay) Hydrogen bonding interactions	[67]
Nanocomposite Zn^2+^@HAP@CS		Electrostatic attraction (positively charged composite)	[68]
CS/PVA/rGO/graphene-based aerogels		Capillary uptake and then chemical adsorption	[101]
DPEA-Cell-OSO^−3^ polymer		Hydrogen bonding	[104]
Nanocomposites Alg/Gel/n-HAP/MNPs		Hydrogen bonding Electrostatic interaction	[28]
Cs/PEG composite membrane			[32]
Composite biopolymer sponge GO-coated PS			[35]
Pop magnetic oak tannin gel (Fe_3_O_4_–OT)			[75]
Polyelectrolyte complexes CS-QSG	Adsorption (heterogeneous multilayer)		[31]
Chitosan derived from mud crab (Scylla serrata) shells adsorbent	Adsorption (monolayer and multilayer)		[112]
AgNPs@Cu@ alginate aerogel composite beads		1. Adsorption of electrons and pollutants on the catalyst’s surface 2. Reduction 3. Desorption on the catalyst’s surface	[29]
Cu-alginate (ZnNPs) hydrogel composite beads		1. Diffuse inside the catalyst’s pore via electrostatic interaction 2. Reduction 3. Desorption on the catalyst’s surface	[47]
Alg/XG/AgNPs/Dex/Ca nanocomposite		1. Electrostatic adsorption of pollutants on the catalyst’s surface 2. Reduction and weaker electrostatic 3. Desorption on the catalyst’s surface	[86]
Nano copper/chitosan aerogel bio composite		1. Adsorption of dyes and BH_4_^-^ ions onto the catalyst’s surface 2. Dye reduction 3. Desorption	[87]
Porous chitosan- gC_3_N_4_ nanosheets		Photodegradation (semiconductor photocatalyst)	[53]
Ag–Co_3_O_4_ NFs/CS-CNFs/SPCE		Electrochemical redox process	[93]
Chitosan-capped zns qds/NiFe_2_O_4_		Enhancement in the absorption of visible light, robust electrostatic interactions, and charge separation of photogenerated charge carriers	[111]
Metal-oxide catalysts MnO*_x_*-PP		Adsorption-enhanced catalytic oxidation	[52]

## Data Availability

Not applicable.
